# Microbial contamination levels in water derived from dental units used in small animal dentistry

**DOI:** 10.3389/fvets.2025.1639712

**Published:** 2025-07-15

**Authors:** Luka Šparaš, Ana Nemec, Majda Biasizzo

**Affiliations:** ^1^Small Animal Clinic, Veterinary Faculty, University of Ljubljana, Ljubljana, Slovenia; ^2^Institute of Food Safety, Feed and Environment, Veterinary Faculty, University of Ljubljana, Ljubljana, Slovenia

**Keywords:** small animal dentistry, dental unit water, microbial contamination, cleaning protocols, biofilm

## Abstract

**Objective:**

This prospective study aimed to assess microbial contamination levels in water from dental units used in small animal dentistry.

**Materials and methods:**

Water from 24 dental units across various clinics in Slovenia was sampled between July 2022 and September 2024. Samples were tested for *Legionella* spp., *Pseudomonas aeruginosa*, *Escherichia coli*, coliform bacteria, intestinal enterococci, and heterotrophic plate counts (HPC) at 36°C. Statistical analysis assessed associations between the water source, implemented disinfection protocols, and microbial contamination levels of water.

**Results:**

A total of 91.3% of the dental units were microbiologically non-compliant when considering potable drinking water standards. When criteria requiring the absence of *Legionella* spp., *P. aeruginosa*, *E. coli*, coliform bacteria, intestinal enterococci, and HPC < 200 colony-forming units (CFU)/ml were applied, 87.0% of the units were non-compliant. When threshold of HPC < 500 CFU/ml was applied, 79.2% of the units remained microbiologically non-compliant. Distilled water supplied 83.3% of units; the remaining 16.7% used municipal water. Disinfection protocols of dental unit waterlines were implemented in 16.7% of the dental units. None of these parameters were statistically significantly associated with microbial contamination levels of the water derived from dental units.

**Conclusions and clinical relevance:**

The high microbial contamination and limited disinfection use in dental units raise concerns about potential health risks to animals and practitioners. This study highlights the need to establish clear guidelines for microbial levels in water derived from dental units used in small animal dentistry, and to assess disinfection protocols in future research.

## 1 Introduction

Dental procedures are frequently performed in small animal veterinary practice, as oral and dental diseases are among the most prevalent health issues in dogs and cats (1–3).

Proper diagnostic procedures and treatments in veterinary dentistry require certain basic equipment, such as a dental unit, radiography and general anaesthesia equipment (4). Dental units in small animal dentistry typically include a compressor, rheostat, and associated instruments such as scalers, a three-way syringe, and high-speed handpieces (5, 6). Dental unit waterlines (DUWLs) consist of a complex network of narrow plastic tubing, serving as the delivery system for water from its source to the dental unit’s associated instruments (7, 8). Microbial contamination of DUWLs is a well-known general problem in dental medicine. Many studies over the past decades have identified high microbial contamination levels [ranging from 10^2^ to 10^8^ colony-forming units (CFU)/ml] in water derived from DUWLs used in human dental practices and the potential health risks for patients and staff (7, 9–11). Microbial contamination of DUWLs can originate from the water source; either from municipal water system or from a water reservoir within the DUWLs. Several structural and operational factors of DUWLs contribute to the rapid formation of biofilm consisting of bacteria, fungi, and protozoa that adhere to DUWLs’ inner surfaces. Structurally, the high surface-to-volume ratio, the use of hydrophobic polymeric plastic tubing with a small diameter, and the laminar flow of water through the system create an ideal environment for biofilm growth. Additionally, operational factors such as prolonged water stagnation further exacerbate these conditions, promoting accelerated biofilm development. The detachment and fragmentation of active biofilm are the primary reservoir for continued microbial contamination within the water supply (7, 8, 12, 13). However, contamination can also result from the absence or malfunction of valves in rotating handpieces, which are designed to prevent the backflow of the patient’s biological fluids caused by negative pressure during the handpiece’s slowdown phase or inappropriate care of the handpieces (7, 12, 14, 15).

According to the recommendation of the US Centers for Disease Control and Prevention (CDC), water from DUWLs used in non-surgical procedures should not contain more than 500 CFU/ml of heterotrophic plate counts (HPCs), consistent with the national drinking water standard (15). In 1996, the American Dental Association (ADA) proposed an even stricter goal of a maximum of 200 CFU/ml of HPCs in such water for the following years (16). However, their current recommendation aligns with that of the CDC (17). In certain countries, there is currently no established maximum limit for CFU/ml of heterotrophic bacteria in water from DUWLs used in human dentistry and there are no standards worldwide for veterinary medicine/dentistry. Existing drinking water legislation in Slovenia states that heterotrophic bacteria levels at 36°C must not exceed 100 CFU/ml. Additionally, these guidelines require that potable water be completely free of *E. coli*, intestinal enterococci and coliform bacteria (18). Sterile saline or sterile water should be used as a coolant/irrigant when performing surgical procedures as per CDC recommendations (15).

To our knowledge, no published studies have examined microbial contamination of water from dental units, or in particular DUWLs, in small animal veterinary practice. Furthermore, no maximum CFU/ml threshold has been established for HPCs in DUWLs water used for veterinary dentistry procedures. The aim of our cross-sectional study is to assess microbial contamination in water from dental units used in small animal veterinary dentistry and to examine its association with the disinfection protocols currently in use.

## 2 Materials and methods

### 2.1 Sampling of the water from dental units

Sampling was conducted from dental units in small animal clinics in Slovenia, where dental procedures are routinely performed and the clinic owners voluntarily participated in the study. Water samples were collected in the morning, prior to the first dental procedure and in accordance with ISO 19458:2006 (the standard for water sampling intended for microbial analysis and water quality assessment) (19). For each dental unit, water samples were collected by the same person (LŠ) from the scaler, high-speed handpiece, and three-way syringe outlets, always in this order and with the dental attachments in place. No flushing of the waterlines was performed prior to sampling. This approach mirrors the methodology used for sampling drinking water directly from a tap, ensuring an accurate evaluation of the water quality as it is consumed—straight from the potentially contaminated source. During sampling, protective measures (i.e., non-sterile nitrile gloves, cap and a face mask) were used to minimize the risks of microbial contamination from the sampler.

A 350 ml sample was taken from each outlet for the determination of HPCs at 22 and 36°C, coliform bacteria, intestinal enterococci, *E. coli* and *P. aeruginosa*. Additionally, a composite 1,000 ml sample was taken by combining equal parts of water from each outlet for the detection of *Legionella* spp.

**Figure 1:**
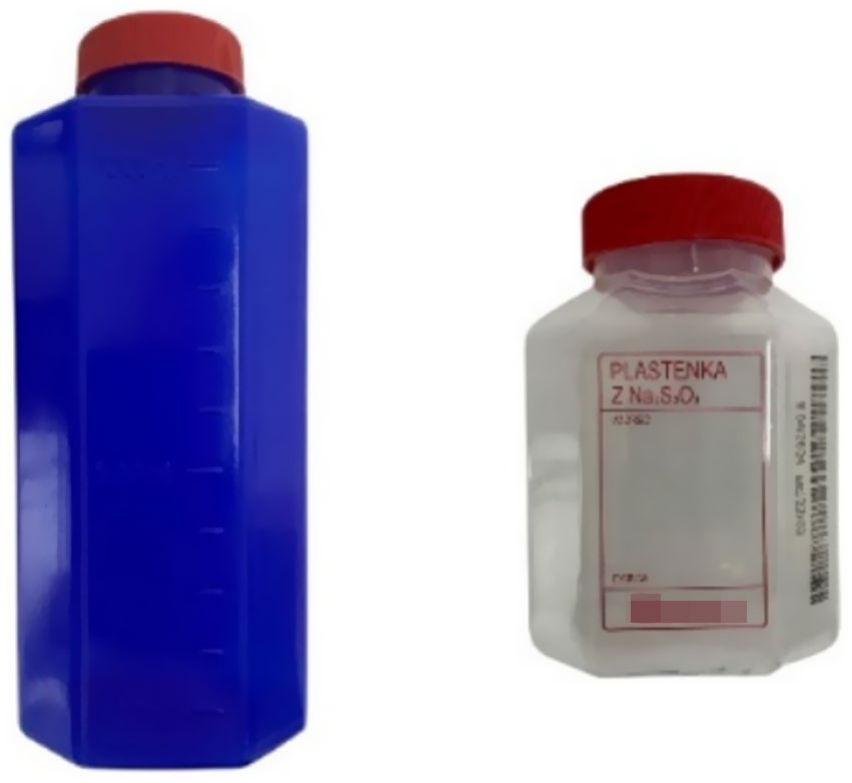
Sterile plastic bottles with sodium thiosulphate solution, used to neutralize free chlorine, were used for water sample collection for microbiological analysis.

Samples were collected in sterile plastic bottles (with sodium thiosulphate solution to neutralize free chlorine; Figure 1) and promptly transported to the laboratory in portable coolers maintained at 4°C. All samples were processed within 24 h for microbiological evaluation.

### 2.2 Microbiological testing

Detection of *E. coli*, coliform bacteria and intestinal enterococci was performed by filtering 100 ml of the sample through 0.45 μm cellulose filters (Millipore, Molsheim, France). The membranes were laid on Cromogenic Coliform Agar (Biokar, Beauvais, France) according to ISO 9308-1:2014 for *E. coli*/coliform growth and on Compass® Enterococcus Agar (Biokar, Beauvais, France) for Enterococcus growth (20). The same procedure was used for detection of *P. aeruginosa* using CN Pseudomonas Gelose/Agar (Biokar, Beauvais, France) according to ISO 16266:2006 (21). The results were expressed as CFU/100 ml.

Heterotrophic plate counts (HPCs) at 36°C and 22°C were determined by the pour plate method in accordance with the ISO 6222:1999 by using Yeast agar (Biolife, Milano, Italy) (22). The results were reported as CFU/ml.

*Legionella* spp. detection was performed in accordance with ISO 11731:2017 (23). Specifically, 0.1 ml of the water sample was directly inoculated onto GVPC agar (Biokar, Beauvais, France). Additionally, 100 ml of the sample was filtered through a cellulose-nitrate membrane (diameter 47 mm, pore size 0.45 μm) (Millipore, Molsheim, France), and the membrane was then placed directly onto GVPC agar. For another 100 ml sample, the filter was overlaid with 20 ml of 0.2 M HCl–KCl buffer (pH 2.2) for 5 ± 0.5 min. After discarding the buffer, the filter was rinsed with 10 ml of sterile water and placed on a third GVPC agar plate. All cultures were incubated at (36 ± 2) °C for 7–10 days, and results were expressed as CFU/L.

### 2.3 Statistical analysis

A chi-square test of independence was used to evaluate potential associations between the presence of bacteria/microbiological suitability and selected factors, including the type of water source, the presence of an implemented cleaning protocol, and the specific output tested (scaler, high-speed handpiece, or three-way syringe outlet). Expected frequencies were computed under the assumption of independence between the variables. The analysis was conducted using SPSS software, which provided the chi-square statistics and the corresponding *p*-value. If the p-value was less than the predetermined significance level (α = 0.05), the null hypothesis was rejected in favour of the alternative hypothesis, suggesting the presence of a statistically significant association between the presence of bacteria/microbiological suitability and the measured factors. In cases where more than 20% of the expected cell frequencies were below 5, Fisher’s exact test (for 2 × 2 contingency tables) or the likelihood ratio test was applied instead of the chi-square test, as these methods were more appropriate for datasets with low expected frequencies. Pairwise deletion was used to handle missing data, allowing for the inclusion of all available cases for each analysis by considering only those variable pairs with non-missing values.

## 3 Results

### 3.1 Dental units

A total of 24 dental units used in small animal dentistry were sampled. The units were supplied with either distilled water from an integrated reservoir (20/24; 83.3%) or municipal water (4/24; 16.7%). Only 16.7% (4/24) of the dental units had implemented any disinfection protocols for their DUWLs (Table 1). In three cases, a disposable cartridge (iM3 Straw, iM3 Inc., Vancouver, WA, USA) containing silver as the active ingredient was used in the unit’s integrated reservoir. The cartridge functioned as a water treatment system, incorporating a weak acid gelular cation exchange resin in its hydrogen form. In one case, a weekly disinfection protocol was implemented for the dental unit using municipal water. As per manufacturer instructions, 6% hydrogen peroxide solution (KaVo OXYGENAL™ 6, KaVo Dental GmbH, Biberach, Germany) was introduced into the DUWLs over the weekend and flushed out before the dental unit was used again. High-speed handpieces were sterilized prior to sampling in five out of 23 cases where they were present on the dental unit. The scaler was sterilized in three out of 24 cases, while the three-way syringe was not sterilized in any case.

**Table 1:** Results of microbiological testing, water source and DUWL cleaning protocol.

Unit	1	2	3	4	5	6	7	8	9	10	11	12
Scaler												
*P. aeruginosa*	~0	~0	>150	~0	~0	23	~0	~0	~0	Mold overgrowth	~0	~0
*E. coli*	~0	~0	33	~0	~0	5	~0	~0	~0	Mold overgrowth	~0	~0
Coliform bacteria	~0	~0	0	~0	~0	8	~0	~0	~0	Mold overgrowth	~0	~0
Intestinal enterococci	~0	~0	0	~0	~0	18	~0	~0	~0	Mold overgrowth	~0	~0
HPCs (22°C)	~0	69	160	~0	270	1,600	30,000	~0	110	Mold overgrowth	>3,000	760
HPCs (36°C)	4,300	35	190	71	43	500	27,000	250	1,600	Mold overgrowth	>3,000	270
Three-way syringe			Not present									
*P. aeruginosa*	~0	~0	/	~0	~0	~0	~0	~0	~0	Mold overgrowth	~0	~0
*E. coli*	~0	~0	/	~0	~0	~0	~0	~0	~0	Mold overgrowth	~0	~0
Coliform bacteria	~0	~0	/	~0	~0	~0	~0	~0	~0	Mold overgrowth	~0	~0
Intestinal enterococci	~0	~0	/	~0	~0	~0	~0	~0	~0	Mold overgrowth	~0	~0
HPCs (22°C)	~0	10	/	~0	~0	120	15	820	490	Mold overgrowth	>3,000	1900
HPCs (36°C)	4,800	410	/	150	500	45	~0	2,000	560	Mold overgrowth	>3,000	2,100
High-speed handpiece			Not present									
*P. aeruginosa*	~0	~0	/	~0	~0	~0	~0	~0	~0	Mold overgrowth	~0	~0
*E. coli*	~0	~0	/	~0	~0	~0	~0	~0	~0	Mold overgrowth	~0	~0
Coliform bacteria	~0	~0	/	~0	~0	~0	~0	~0	~0	Mold overgrowth	~0	~0
Intestinal enterococci	~0	~0	/	~0	~0	~0	~0	~0	~0	Mold overgrowth	~0	~0
HPCs (22°C)	8	61	/	~0	36	~0	8	11,000	450	Mold overgrowth	>3,000	>3,000
HPCs (36°C)	4,600	220	/	170	280	~0	~0	420	550	Mold overgrowth	>3,000	>3,000
*Legionella* spp.	~0	~0	~0	~0	~0	~0	~0	~0	~0	~0	~0	~0
Water source	Distilled	Distilled	Municipal	Distilled	Distilled	Distilled	Distilled	Distilled	Distilled	Distilled	Distilled	Distilled
DUWLs cleaning protocol	No	No	No	Yes	No	No	No	No	No	No	Yes	No

Unit	13	14	15	16	17	18	19	20	21	22	23	24
Scaler												
*P. aeruginosa*	~0	~0	~0	~0	~0	~0	~0	~0	~0	>150	~0	~0
*E. coli*	~0	~0	~0	~0	~0	~0	~0	~0	>150	~0	~0	~0
Coliform bacteria	~0	~0	~0	~0	~0	~0	~0	~0	1	~0	~0	~0
Intestinal enterococci	~0	~0	~0	~0	~0	~0	~0	~0	1	~0	~0	~0
HPCs (22°C)	460	>3,000	~0	>30,000	~0	>3,000	~0	3,000	>30,000	13,000	2,500	200
HPCs (36°C)	1,200	>3,000	~0	>30,000	~0	920	~0	1,100	>30,000	16,000	4,800	1,000
Three-way syringe	Not present											
*P. aeruginosa*	/	~0	~0	~0	~0	~0	~0	~0	>150	~0	~0	~0
*E. coli*	/	~0	~0	~0	~0	~0	~0	~0	36	~0	~0	~0
Coliform bacteria	/	~0	~0	~0	~0	~0	~0	~0	>150	8	~0	~0
Intestinal enterococci /		~0	~0	~0	~0	~0	~0	~0	~0	3	~0	~0
HPCs (22°C)	/	1,100	~0	>30,000	730	160	~0	21	6,000	86	35	67
HPCs (36°C)	/	~0	~0	>30,000	1,100	420	~0	70	>30,000	150	500	310
High-speed handpiece												
*P. aeruginosa*	~0	~0	~0	~0	~0	~0	~0	~0	4,000	~0	41	~0
*E. coli*	~0	~0	~0	~0	~0	~0	~0	~0	7	~0	~0	~0
Coliform bacteria	~0	~0	~0	~0	~0	~0	~0	~0	~0	~0	~0	~0
Intestinal enterococci	~0	~0	~0	~0	~0	~0	~0	~0	~0	~0	~0	~0
HPCs (22°C)	1,500	>3,000	~0	>30,000	3,500	750	~0	520	24,000	25,000	650	1700
HPCs (36°C)	2,100	>3,000	~0	>30,000	10,000	260	~0	760	>30,000	4,300	1,600	1,000
*Legionella* spp.	~0	~0	~0	~0	~0	~0	~0	~0	~0	~0	~0	~0
Water source	Distilled	Municipal	Distilled	Distilled	Distilled	Distilled	Distilled	Municipal	Distilled	Distilled	Distilled	Municipal
DUWLs cleaning protocol	No	No	No	No	No	No	Yes	Yes	No	No	No	No

~0 – under method detection limit.

### 3.2 Microbiological testing results

Detailed results of the microbiological testing are available in Table 1.

Water from one unit was microbiologically unsuitable because of mold growth, what also prevented the assessment of HPCs and other bacteria. This unit was deemed microbiologically non-compliant but could not be classified within the predefined categories and was therefore excluded from statistical analysis.

In the overall assessment of the microbial quality of water from the dental units, we considered the presence of *Legionella* spp., *P. aeruginosa, E. coli*, coliform bacteria, intestinal enteroccoci, and different thresholds of HPCs at 36°C: HPC < 100 CFU/ml, HPC < 200 CFU/ml, and HPC < 500 CFU/ml. Under the most stringent criterion used for potable drinking water in Slovenia (absence of bacteria and HPC at 36°C < 100 CFU/ml) 91.3% of the dental units were microbiologically non-compliant. When applying the former ADA criterion of absence of bacteria and HPC < 200 CFU/ml, 87.0% of sampled dental units were microbiologically non-compliant. Under the least restrictive CDC criterion, with absence of bacteria and HPC < 500 CFU/ml, 79.2% of the dental units were found to be microbiologically non-compliant. HPC values at 36°C ranged from approximately 0 (below the detection limit) to over 30,000 CFU/ml (above the quantification limit). Two of the dental units had been in use for less than 6 months; one met the microbiological standard under the criterion of absence of bacteria and HPC < 200 CFU/ml, while the other failed to comply with any of the applied criteria.

At the level of the individual tested outlets, and applying the most stringent standards for potable water, 73.9% of samples from the scaler, 71.4% from the three-way syringe, and 81.8% from the high-speed handpiece were microbiologically non-compliant. When considering the least restrictive CDC criterion, 56.6% of samples from the scaler, 38.1% from the three-way syringe, and 59.1% from the high-speed handpiece were microbiologically non-compliant. HPCs values at 36°C ranged from approximately 0 (below the detection limit) to over 30.000 CFU/ml (above the quantification limit) across all three sampled outlets.

*P. aeruginosa* was detected in five dental units. At the level of the individual tested outlets, it was found in 13% of samples from the scaler, 4.8% from the three-way syringe, and 9.1% from the high-speed handpiece. *E. coli* was present in three dental units, occurring in 13% of samples from the scaler, 4.8% from the three-way syringe, and 4.5% from the high-speed handpiece. Coliform bacteria were detected in three dental units, found in 8.7% of samples from the scaler, 9.5% from the three-way syringe, and none from the high-speed handpiece. Intestinal enteroccoci were also detected in three dental units, found in 8.7% of samples from the scaler, 4.8% from the three-way syringe, and none from the high-speed handpiece.

*Legionella* spp. was not detected in any of the samples.

Statistical analysis revealed no significant association between the type of water source used in dental units and the microbial suitability of tested water samples across the evaluated criterion: HPC < 100 CFU/ml at 36°C (*p* = 1.00), HPC < 200 CFU/ml at 36°C (p = 1.00), and HPC < 500 CFU/ml at 36°C (*p* = 0.539).

Similarly, the association between the implemented cleaning and disinfection protocol for dental unit waterlines and microbiological suitability of the water from the dental units was not statistically significant: HPC < 100 CFU/ml at 36°C (*p* = 0.324), HPC < 200 CFU/ml at 36°C (*p* = 0.067), and HPC < 500 CFU/ml at 36°C (*p* = 0.194).

No statistically significant association was found between the different tested water outlets (scaler, high-speed handpiece, and three-way syringe) and the microbiological suitability of the water, for both thresholds HPC < 100 CFU/ml at 36°C (*p* = 0.706) and HPC < 500 CFU/ml at 36°C (*p* = 0.325).

## 4 Discussion

The high levels of microbial contamination found in water derived from dental units used in small animal dentistry in this study align with previous research in human dentistry, which identified contamination levels as high as 10^8^ CFU/ml (24–27). Only 8.7% of the dental units in this study met the national criterion for drinking water quality. Additionally, 13% of the dental units met ADA’s former recommendation for maximal levels of HPCs (< 200 CFU/ml), while 20.8% met the recommendation from the CDC (< 500 CFU/ml). These recommendations are intended for non-surgical dental treatments in humans, whereas in veterinary dentistry, dental units are often used in procedures requiring surgical tooth extractions. In one of the 24 dental units in this study, mold growth was observed, preventing assessment of bacterial presence and contamination levels.

Water plays a crucial role in dental treatment, serving both as a coolant for instruments (thereby preventing iatrogenic damage to oral and dental tissues) and as an irrigant for surgical field. In addition to direct contact with water, both dental staff and patients are exposed to aerosols generated by high-speed rotating instruments or ultrasonic and sonic scalers (27–30). Aerosols can contain microorganisms and can remain airborne for extended periods, allowing them to spread throughout the dental room (7, 31–33). Substantial microbial contamination linked to the use of high-speed rotating instruments was recorded more than 1.5 meters from the patient and may be associated with occupational health risks including high risks for asthma (34–37). In human dentistry, various strategies are employed to mitigate aerosol exposure in dental settings and one of the most effective ways to minimize aerosol exposure during dental procedures is to capture aerosols directly at the source—the patient’s mouth. This can be achieved using source control methods, such as high-volume evacuation and alternative dental evacuation systems (38–40). To the authors’ knowledge, veterinarians performing dentistry procedures in small animals generally rely on basic water/saliva ejectors, which are also not consistently used.

The selection of specific microbes (HPC, *E. coli*, coliform bacteria and intestinal enterococci) for testing was based on the national standard for potable water in Slovenia (18). Presence of other microbes (*P. aeruginosa, Legionella* spp.) was additionaly tested due to the known potential of these bacteria to contaminate medical equipment and their (opportunistic) pathogenicity (7–10, 14). *Legionella* spp. testing was initially conducted using a composite sample collected from individual outlets of the dental unit, with the aim of determining the presence of the bacteria within particular parts equipment only if the whole sample was found contaminated.

*Legionella* spp. was not detected in any of the samples obtained in this study. Although this Gram-negative bacterium has frequently been reported in previous studies of microbial contamination in DUWLs, the reported prevalence varies widely (41–43). The inhalation of aerosols contaminated with *Legionella* spp. can pose serious health risks to humans, as it can result in Legionnaires’ disease, a severe form of pneumonia (44–46). Dental units provide an ideal environment for the multiplication and potential transmission of *Legionella* spp., with documented cases of legionellosis in a patient acquired through contaminated dental units (42, 47, 48). The occupational risk of *Legionella* spp. infection among dental health workers has also been widely discussed (14, 43, 49–52). The absence of *Legionella* spp. in dental units in our study may be attributed to the fact that only four out of 24 sampled dental units used municipal water as their source, while the others relied on distilled water from small reservoir bottles integrated into the dental units. Previous studies have shown that large institutions are more susceptible to *Legionella* spp. colonization due to the use of large water storage tanks (53). The prevalence of *Legionella* spp. may also vary depending on the geographical location (43, 52).

In our study, *P. aeruginosa* was detected in five out of 23 dental units. The presence of *P. aeruginosa* in DUWLs has often been the subject of research due to its pathogenic role in susceptible human hosts. Some of the previous studies have reported the prevalence of *P. aeruginosa* in water from DUWLs used in human dental practices to range from 5 to 24% (25, 54–56). To our knowledge no prior published studies have identified the prevalence of *P. aeruginosa* in DUWLs used in small animal dentistry. Although *P. aeruginosa* is considered one of the most important human pathogens in hospital-acquired infections, there are not many reports linking *Pseudomonas* spp. infections directly to patient exposure to contaminated DUWLs (55). *P. aeruginosa*, as in humans, is recognized as an opportunistic pathogen in dogs and cats associated with ulcerative keratitis, otitis, pyoderma, urinary tract infections, wound and respiratory tract infections in both species (57–61). There is currently no information regarding dental units as a potential source of *P. aeruginosa* infection in small animals. *P. aeruginosa* warrants significant attention due to its high levels of antimicrobial resistance. Notably, in the WHO Bacterial Priority Pathogen List 2024, it has been reclassified from the critical to the high-priority group. Organisms in this group are significantly difficult to treat, cause a substantial disease burden, show increasing trends in resistance, are uniquely challenging to prevent, are highly transmissible, and have few potential treatments under development (62).

*E. coli* was detected in three of the dental units in this study. This bacterium is commonly used as an indicator of water quality because its presence signals fecal contamination of the water (63). Consequently, this contamination raises the likelihood that enteric pathogens may also be present. In two of the units where *E. coli* was detected, coliform bacteria and enterococci were also identified.

Cross-transmission of microorganisms is a common occurrence in dental treatment, primarily due to direct or indirect contact with patients, dental staff, and contaminated aerosols. While the overall risk of infection remains low, it is a greater concern when treating immunocompromised patients and the results of this study warrant further research to assess the risk to animals, as its potential pathological impact cannot be overlooked (12, 14).

Rapid biofilm formation on devices used in medical and dental procedures is a well-documented public health problem that is difficult to address as complex biofilm becomes very resistant (64–70). Once mature, biofilm can act as a reservoir for microorganisms that are continuously released into the water from DUWLs during biofilm detachment and fragmentation (7, 41, 69). As the aim of this study was to evaluate the overall microbial burden from water derived from the entire dental unit system, water quality of the source (input water) was not tested separately and the water samples were obtained with the dental attachments in place (where most of these were not sterilized). Therefore, it is not possible to ascribe the poor water quality derived from dental units in this study solely to DUWLs biofilm formation. However, as substantial variations in water quality were observed between different outlets of the same dental unit, biofilm formation within parts of DUWLs likely contributed to the microbial contamination.

Moreover, only four out of 24 dental units had a protocol in place for cleaning and disinfecting DUWLs. Notably, only two of these four units met the CDC and ADA recommendations for HPCs, and just one met national potable water standards. As this study only recorded whether a cleaning and disinfection protocol was present or absent, without evaluating its’ specific procedures, the reasons why microbial contaminations levels were high in the other two dental units remain unclear. Also, there was no statistically significant difference in microbial water quality between dental units with and without a cleaning and disinfection protocol. This may be due to the small sample size, as several prior studies in human dentistry have demonstrated that the use of chemical disinfectants significantly reduces microbial contamination in DUWLs. Protocols using peracetic acid, ethylenediaminetetraacetic acid (EDTA) tetrasodium salt, sodium hypochlorite, chlorhexidine gluconate, iodine povidone, hydrogen peroxide and chlorine dioxide have been studied. Various dental chair manufacturers specify products that are compatible with their equipment to prevent damage (41, 71–76). Additional measures, such as cleaning, oiling and autoclaving handpieces (which was rarely observed during our study and likely contributed to the observed contamination of the water sampled), regularly replacing them, and flushing the waterlines between patients for 20 to 30 s, also play a crucial role in controlling water contamination levels (15, 76).

## 5 Conclusion

The poor microbial quality of the water derived from dental units in this study, combined with the general absence of cleaning and disinfection protocols for DUWLs and lack of sterilization of handpieces, highlights a significant concern. Given that distilled water was used in 83.3% of the sampled cases (minimizing the likelihood of contamination at the water source) there is a clear need to implement effective disinfection measures for DUWLs and dental instruments in veterinary dentistry. Additionally, guidelines should be established to define the maximum recommended levels of microbial contamination in DUWL water, similar to those in human dentistry, along with regular monitoring to ensure compliance. Further studies with larger sample sizes, sampling of the input water, expanding microbiological testing to include other microorganisms such as mycobacteria and free-living protozoa, and an evaluation of the effectiveness of different DUWLs disinfection protocols in small animal dentistry are warranted.

## Data Availability

The raw data supporting the conclusions of this article will be made available by the authors, without undue reservation.
